# The Role of Satisfaction With Job and Cognitive Trauma Processing in the Occurrence of Secondary Traumatic Stress Symptoms in Medical Providers Working With Trauma Victims

**DOI:** 10.3389/fpsyg.2021.753173

**Published:** 2022-01-06

**Authors:** Piotr Jerzy Gurowiec, Nina Ogińska-Bulik, Paulina Michalska, Edyta Kȩdra, Aelita Skarbalienė

**Affiliations:** ^1^Institute of Health Sciences, University of Opole, Opole, Poland; ^2^Department of Health Psychology, Institute of Psychology, University of Lodz, Łódź, Poland; ^3^State Higher Vocation School in Glogow, Medical Institute, Glogow, Poland; ^4^Faculty of Health Sciences, Klaipeda University, Klaipėda, Lithuania

**Keywords:** STS symptoms, job satisfaction, cognitive trauma processing, medical providers working with trauma victims, secondary trauma

## Abstract

**Introduction:** As an occupational group, medical providers working with victims of trauma are prone to negative consequences of their work, particularly secondary traumatic stress (STS) symptoms. Various factors affect susceptibility to STS, including work-related and organizational determinants, as well as individual differences. The aim of the study was to establish the mediating role of cognitive trauma processing in the relationship between job satisfaction and STS symptoms among medical providers.

**Procedure and Participants:** Results were obtained from 419 healthcare providers working with victims of trauma (218 nurses and 201 paramedics). Three questionnaires, namely the Secondary Traumatic Stress Inventory, Work Satisfaction Scale, and Cognitive Trauma Processing Scale, were used in the study, as well as a survey developed for this research. Correlational and mediation analyses were applied to assess relations between variables.

**Results:** The results showed significant links between STS symptoms and both job satisfaction and cognitive processing of trauma. Three cognitive coping strategies play the intermediary role in the relationship between job satisfaction and symptoms of secondary traumatic stress. However, this role varies depending on preferred strategies.

**Conclusion:** Nurses and paramedics are significantly exposed to the occurrence of STS. Thus, it is important to engage health care providers in activities aimed at preventing and reducing symptoms of STS.

## Introduction

### Secondary Traumatic Stress in Medical Personnel Working With Trauma Victims

The term secondary traumatic stress (STS), or secondary traumatic stress disorder (STSD), was adopted by Figley (1995), who characterized STS as stress associated with helping trauma victims or people in crisis. STS is described as the emotional and behavioral consequences experienced by an individual after getting an information about another person’s stressful or traumatic situation. As medical providers are direct contact with people who suffer from injuries, accidents, and illness, STS may be identified as a risk factor in medical providers (Beck, 2011). It has been argued that the symptoms of STS belong to the four categories related to post-traumatic stress disorder (PTSD): intrusion, avoidance, negative alterations in cognitions and mood, alterations in arousal and reactivity (American Psychiatric Association, 2013). Some differences remain, however, primarily from the source of trauma; PTSD symptoms manifest as a consequence of direct exposure to trauma, whereas STS symptoms manifest as a result of indirect traumatic experiences. The DSM criterion for traumatic stressors, i.e., exposure to aversive elements of traumatic events in the course of work, may indicate that PTSD and STSD are the same phenomenon (Greinacher et al., 2019). Nevertheless, most researchers point out the need to distinguish between these two constructs (McCann and Pearlman, 1990; Figley, 1995, 1999; Crumpei and Dafinoiu, 2012; Nimmo and Huggard, 2013; Michael et al., 2016; Missouridou, 2017; Greinacher et al., 2019). It is worth noting that negative outcomes of secondary exposure to trauma may also be described as Compassion Fatigue (CF) or Vicarious Traumatization (VT) (McCann and Pearlman, 1990; Figley, 1995, 1999).

The literature confirms the high occurrence of STS symptoms among medical providers, especially nurses (Dominguez-Gomez and Rutledge, 2009; Hooper et al., 2010; Missouridou, 2017; Al-Majid et al., 2018; Mottaghi et al., 2019; Ogińska-Bulik and Michalska, 2020b; Ortega-Campos et al., 2020; Salimi et al., 2020; Xie et al., 2020; Ogińska-Bulik et al., 2021). For example, according to one report, secondary traumatic stress criteria were met by 64% of nurses working in Irish emergency medical services (Duffy et al., 2015). Likewise, just over half of Jordanian emergency nurses presented severe or high STS level (Ratrout and Hamdan-Mansour, 2020). However other data show that STS was present in only 7% of nurses caring for trauma patients (Von Rueden et al., 2010). Such differences can be the result of the measurement tools.

One study found that nearly 13% of American paramedic personnel meet STSD criteria (Roden-Foreman et al., 2017). Symptoms of STS among ambulance personnel were also revealed by Argentero and Setti (2011). The studies conducted in Poland support the findings about high risk of secondary traumatic stress disorder occurrence in medical health providers, in both nurses and paramedics (Ogińska-Bulik and Juczyński, 2020). The obtained results indicated that among five professional groups (nurses, paramedics, therapists, probation officers, and social workers) the highest severity of secondary traumatic stress symptoms was revealed by healthcare providers. Furthermore, nearly 50% of both nurses and paramedics working on oncology or posttraumatic units showed a high likelihood of STSD occurrence.

### The Role Satisfaction With Work and Cognitive Processing of Trauma in the Occurrence of Secondary Traumatic Stress Symptoms

Although many studies have investigated the link between type of work and STS, relatively few have assessed the connections between job satisfaction and secondary traumatic stress symptoms. Furthermore, among those that have, the majority focused on satisfaction gained from helping, which may be treated as an opposition to CF (Sodeke-Gregson et al., 2013), rather than the general feeling of job satisfaction. The study of nurses from America showed the negative correlation between symptoms of STS and helping satisfaction, CF, or burnout (Hinderer et al., 2014). Similarly, low level of satisfaction with job and increased working hours were linked to STS and explain almost 10% of the variance among Chinese nurses (Wang et al., 2020). Other research indicated that nursing personnel with better satisfaction with work revealed a lower level of CF than their less satisfied co- workers (Kelly et al., 2015). Some authors (Kelly and Lefton, 2017) showed that satisfaction with job was a significant predictor for STSD, reducing the severity of STS symptoms and increasing compassion satisfaction in nurses from critical care.

Some negative associations of secondary traumatic stress symptoms and job satisfaction has been showed among other groups of professionals as well. For example, job satisfaction turned out to play a role of mediator in the relationship between secondary traumatic stress and work engagement among specialists who consult people addicted to substances (Bride and Kintzle, 2011). The authors stressed that job satisfaction, as one of the main predictors of work engagement, may play an important role in determining who is most susceptible to STS. Interestingly, some research failed to confirm the link between work satisfaction and symptoms of STS, both among professionals who help trauma victims (Ogińska-Bulik and Juczyński, 2020) and in nurses (Balinbin et al., 2020). Some studies even stressed an inverse relationship, with STS leading to dissatisfaction with job (Kleis and Kellog, 2020). The insufficient research on this subject and inconclusive results indicate the need for further research.

Another important factor for the development of STSD is cognitive trauma processing (McCann and Pearlman, 1990; Dutton and Rubinstein, 1995; Ogińska-Bulik and Juczyński, 2020). Since STSD and PTSD symptoms show significant similarities, the phenomenon of secondary traumatization may be explained by models used to describe PTSD. These include the cognitive shattered assumptions theory (Janoff-Bulman, 1989), the cognitive model of PTSD (Ehlers and Clark, 2000), and the emotional processing model (Foa and Kozak, 1991). The post-traumatic disorders in these models are treated as errors in the cognitive trauma processing that lead to distorted beliefs about the oneself and world. The Constructivist Self-Development Theory (McCann and Pearlman, 1990) and the Ecological Framework of Trauma (Dutton and Rubinstein, 1995) refer to the cognitive approach in connection to secondary traumatization. Additionally, the Ecological Framework of Trauma emphasizes the role of environmental factors, especially work-related, including job satisfaction, in the STS development.

The purpose of cognitive trauma processing is to make meaning of the events experienced and to adapt to a new reality altered by the trauma experienced. In order to make meaning, restore or extend the level of previous psychological functioning, the individual makes certain cognitive efforts. Williams, Davis, and Millsap indicated as manifestations of effective trauma processing the reduction of levels of negative emotions (especially in the form of guilt or shame), the incorporation of information about the traumatic event and its acceptance, the perception of some positive sides of the trauma, and desensitization, which involves the reduction of negative emotions and stress. This cognitive processing often takes the form of cognitive coping strategies: downward comparison, positive cognitive restructuring, resolution/acceptance, regret, and denial.

To date, very few studies have assessed the relationship between cognitive processing in the form of cognitive strategies and STS. One such study was conducted in 5 groups of specialists who worked with victims of trauma; findings confirmed the relationship between cognitive coping strategies and STS symptoms (Ogińska-Bulik and Juczyński, 2020). Regret and denial had the strongest positive links with STS symptoms; however, this relationship requires further investigation among medical personnel. Research suggests that, among paramedics, deteriorated strategies of intrusion coping and dysfunctional beliefs play a role of predictor for PTSD and STSD (Michael et al., 2016).

### Aim of the Research

The current research aim was to examine the links between job satisfaction, cognitive trauma processing, and symptoms of secondary traumatic stress among healthcare providers exposed to secondary trauma, as well as the mediating role of cognitive processing of trauma in the relation betwixt job satisfaction and STS. The indicators of cognitive trauma processing were cognitive coping strategies.

The adopted model referred to cognitive theories of trauma, in particular to the model of PTSD developed by Ehlers and Clark (2000) and the concept developed by Foa and Kozak (1991) According to these models, an individual’s cognitive activity contributes significantly to the formation and maintenance of trauma negative consequences. Moreover, Ehlers and Clark (2000) believe that the cognitive processing of trauma is the mechanism that explains the negative effects incurred by the individual and may play the role of mediator. Dutton and Rubinstein (1995) Ecological Framework of Trauma model explains secondary trauma in terms of the individual’s coping strategies and workplace environmental factors, including job satisfaction. We hypothesized the following: (1) both job satisfaction and cognitive coping strategies would correlate with STS symptoms, (2) job satisfaction would be associated with cognitive coping strategies, (3) medical providers working with trauma victims would be satisfied with their work and engage in cognitive trauma processing expressed as coping strategies, (4) these strategies, especially the positive ones, would decrease the severity of STS symptoms, creating a pathway from job satisfaction to secondary posttraumatic negative changes.

### Method^1^

The study included 430 participants who mostly work with injured individuals. Their participation was anonymous and voluntary. The participants were recruited mainly from polish hospitals: emergency, intensive care, oncology wards, and hospice units between 2019 and 2020. The study obtained approval from the bioethics committee from Opole (no 81/P1/2019). Participants were initially required to provide verbal consent; completion of the study questionnaire evidenced formal written consent. Authors or individual designated by the authors delivered the questionnaires to the participants’ workplace, where they were completed while participants were on duty. Practice as a nurse or paramedic and regular interactions with people experiencing traumatic events, such as an accident or a life or health threatening illness (stroke, heart attack, cancer) were the inclusion criteria. The analysis excluded 11 subjects due to missing data, leaving 419 individuals aged 19–65 years (*M* = 39.6, *SD* = 11.03); 137 (32.7%) males and 282 (67.3%) females. The study group consisted of paramedics (*n* = 201), of whom 60.2% were men, and nursing personnel (*n* = 218) of whom 92.7% were women. Most of the paramedics helped people who experienced various types of accidents, mainly car accidents (57.2%) and after injuries such as heart attacks and strokes (42.8%). The nursing team consisted of individuals who worked with oncology patients (87.7%) and car accident victims (18.3%). Work experience ranged from 1 to 43 years (M =12.18, SD = 9.74), the number of hours per week devoted to assisting injured patients ranged from 2 to 90 (*M* = 38.64, SD = 15.64), the workload in the form of percentage of work devoted to assisting patients directly in relation to all work, ranged from 2 to 100% (*M* = 69.11, SD = 31.89). Three standardized questionnaires were used in the study.

**The Secondary Traumatic Stress Inventory (STSI)** is a Polish adaptation of PCL–5 developed by Weathers et al. (2020) and conducted by Ogińska-Bulik and Juczyński (2020). It is a self- assessment method used for testing people who helping victims of trauma. Similar to the PCL–5, it consists of 20 statements about traumatic events (e.g., “Repeated, disturbing, and unwanted memories of the stressful experience”) concerning the symptoms from four criteria of PTSD: (1) intrusion, (2) avoidance (3) negative alterations in cognitions and mood (4) alterations in arousal and reactivity. The modified version of the questionnaire linked the 20 items to the help provided to trauma victims. For example, the phrase “of my patients” was added to some statements. Individuals were asked the extent to which they had experienced these reactions in the past month, due to the assistance they provided, on a 5–point scale. The Cronbach’s alpha coefficient for STSI was 0.90 and for each factors: 0.71; 0.85; 0.89; 0.87.

**The Work Satisfaction Scale (WSS)** is a Diener et al. (1985) satisfaction with Life Scale version, modified by Zalewska, 2003. Originally designed to evaluate general life satisfaction, the modified statements refer to the evaluation of work (e.g., “In many aspects my work is almost perfect”). Participants indicated the extent to which they agreed with each statement on a 7-point scale. All items are a part of one dimension. The Cronbach’s alpha indicator was 0.86.

**The Cognitive Processing of Trauma Scale (CPOTS)** developed by Williams et al. (2002) was adapted to Polish conditions by Ogińska-Bulik and Juczyński (2018) and adjusted for the study of people indirectly exposed to trauma. The questionnaire contains 17 items (e.g., “Overall, there is more good than bad in this experience”) and assess cognitive processing elements: downward comparison, positive cognitive restructuring, resolution/acceptance, denial, and regret. Participants answer the items on a 7-point scale. The coefficients obtained were: 0.82 for resolution/acceptance, 0.89 for downward comparison, 0.84 for positive cognitive restructuring, 0.72 for regret, and 0.56 for denial.

### Statistical Procedures

Data analyses were conducted using 25 version of IBM SPSS. Initially we calculated means, standard deviations, and Pearson’s r to check the relationships between the variables. Then we used the PROCESS by Preacher and Hayes (2008) for mediation analyses. Job satisfaction played a role of independent variable and predictor, cognitive coping strategies were mediators and STS symptoms were outcome and dependent variables.

## Results

Table 1 presents the main statistics for variables (means, standard deviations and Pearson’s r). The mean STS symptom score of 31.00 (SD = 19.59) was slightly higher than the average score (*M* = 24.14, SD = 16.11) obtained from specialists who work with victims of trauma (Ogińska-Bulik and Juczyński, 2020). In total, 237 individuals (56.6%) scored below 33, indicating a low probability of developing STSD. Conversely, high level of secondary traumatic stress symptoms, implying a high probability of STSD diagnosis, was recorded in 182 participants (43.4%).

**TABLE 1:** Descriptive statistics of analyzed variables (*N* = 419).

Variables	1	2	3	4	5	6	7	8	9	10	11
1. STS total	−										
2. STS.1	0.908***	−									
3. STS.2	0.832***	0.771***	−								
4. STS.3	0.963***	0.815***	0.759***	−							
5. STS.4	0.945***	0.785***	0.715***	0.886***	−						
6. Job satisfaction	−0.401***	−0.355***	−0.322***	−0.399***	−0.376***	−					
7. CPOT.1	−0.170**	−0.150**	−0.098*	−0.184**	−0.156**	0.355***	−				
8. CPOT.2	−0.040	−0.047	−0.045	−0.017	−0.050	0.180**	0.587***	−			
9. CPOT.3	−0.320***	−0.289***	−0.248***	−0.310***	−0.307***	0.405***	0.674***	0.510***	−		
10. CPOT.4	0.175**	0.150**	0.152**	0.197**	0.137**	0.108*	0.429***	0.627***	0.333***	−	
11. CPOT.5	0.181**	0.158**	0.136**	0.195**	0.157**	0.139**	0.393***	0.474***	0.289***	0.695***	−
Mean	31.00	7.98	3.26	10.22	9.55	21.28	8.66	8.14	12.20	8.61	6.42
Standard deviation	19.59	4.93	2.28	7.35	6.51	6.65	4.23	4.43	5.46	5.22	4.15

STS, secondary traumatic stress; STS.1, intrusion; STS.2, avoidance; STS.3, negative alterations in cognitions and mood; STS.4, alterations in arousal and reactivity; CPOT, cognitive processing of trauma; CPOT.1, positive cognitive restructuring; CPOT.2, downward comparison; CPOT.3, resolution/acceptance; CPOT.4, denial; CPOT.5, regret. *p < 0.05; **p < 0.01; ***p < 0.001 (Two-tailed).

The STS score obtained by nurses (*M* = 32.23, *SD* = 20.69), although slightly higher, did not differ significantly from the STS score obtained by paramedics (*M* = 29.67, *SD* = 18.28). The percentage of individuals at high risk for STSD was similar across groups: 43.3% among paramedics and 43.6% among nursing staff. Gender did not influence STS symptom severity (males: *M* = 30.32, *SD* = 18.31; females: *M* = 31.33, *SD* = 20.20, *t* = −0.49). Gender also did not differ the severity of STS in particular groups of professionals.^2^ There was a positive, although weak, association between participants’ age, and STS symptoms (*r* = 0.12, *p* < 0.05). STS symptoms were negatively correlated with number of hours worked per week (*r* = −0.21, *p* < 0.001) and workload (*r* = −0.12, *p* < 0.01), indicating that the more hours worked per week and the greater the workload, the lower the severity of STS symptoms. Seniority as a paramedic/nurse did not significantly correlate with overall STSI score.

Job satisfaction negatively correlated with all four STS symptom criteria. The highest correlation coefficient value was obtained for negative changes in the cognitive and emotional domains, while the lowest was obtained for avoidance. Job satisfaction was positively correlated with all cognitive trauma coping strategies. Significantly higher values of correlation coefficients applied to positive strategies, mainly positive cognitive restructuring and resolution/acceptance, than to negative ones.

Cognitive trauma processing, as captured by cognitive coping strategies, was also associated with STS symptoms. The positive correlation existed between negative strategies, i.e., denial and regret and STS symptoms, while negative correlations were between positive strategies, i.e., positive cognitive restructuring and resolution/acceptance and STS symptoms. In contrast, the downward comparison strategy was not significantly correlated with STS symptom severity. However, the obtained values of correlation coefficients for these variables are lower than for the correlation between job satisfaction and STS.

**FIGURE 1:**
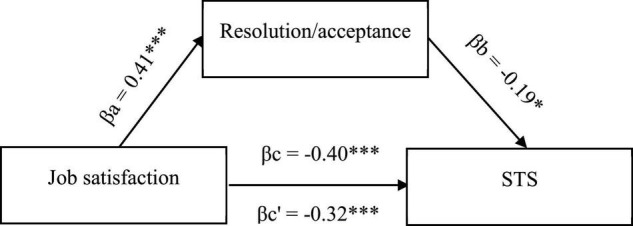
Model of associations between job satisfaction, cognitive coping strategy in the form of resolution/acceptance, and STS. βa,b, indirect effect; βc, total effect; βc’, direct effect; *p < 0.05; **p < 0.01; ***p < 0.001.

**FIGURE 2:**
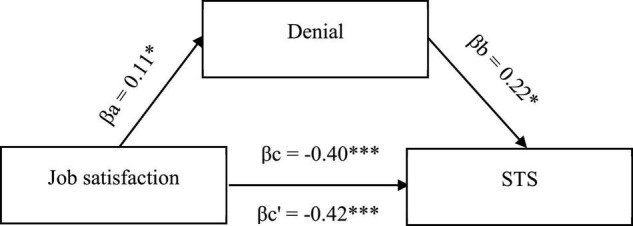
Model of associations between job satisfaction, cognitive coping strategy in the form of denial, and STS. βa,b, indirect effect; βc, total effect; βc’, direct effect; *p < 0.05; **p < 0.01; ***p < 0.001.

**FIGURE 3:**
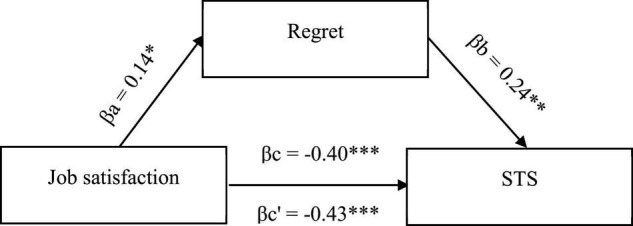
Model of associations between job satisfaction, cognitive coping strategy in the form of regret, and STS. βa,b, indirect effect; βc, total effect; βc’, direct effect; *p < 0.05; **p < 0.01; ***p < 0.001.

The existing relationships between STS, job satisfaction, and cognitive trauma processing substantiates the search for more complex associations, including cognitive processing of trauma as mediators. Hence, to confirm that the mechanism underlying the occurrence of secondary negative changes is based on cognitive activity, mediation analysis was conducted to check the mediating role of cognitive coping strategies in the relationship between job satisfaction and STS symptoms. Three significant models, taking into account three cognitive coping strategies, were obtained (Figures 1–3).

As shown in Figure 1, job satisfaction was a negative predictor of STS symptoms and a positive predictor of the resolution/acceptance strategy, which in turn protects against STS symptoms (negative correlation). Upon the introduction of this strategy as a mediator in the correlation between job satisfaction and symptoms of STS, the strength of the negative correlation between the variables is reduced (partial mediation). That is, job satisfaction interacts with STS symptoms both directly and indirectly through a resolution/acceptance strategy, reducing the likelihood of secondary negative posttraumatic changes.

Two negative strategies were also shown to play a role of mediators for relationship between job satisfaction and STS symptoms, namely, denial (Figure 2) and regret (Figure 3). Both promote STS symptoms while being positively correlated with job satisfaction. In this case, however, the effect of partial suppression could be observed, meaning the use of these strategies increased, albeit slightly, the strength of the association between job satisfaction and STS symptoms. This relationship was still negative, suggesting that medical providers who are satisfied with their work while feeling grief over patients’ pain and suffering are less likely to experience STS symptoms than those who are satisfied with their jobs but do not feel grief and do not use denial. No significant correlations were found between the remaining coping strategies.

## Discussion

We found that 43.4% of the surveyed medical providers working with trauma victims were at high risk of STSD development, a finding that is consistent with the literature (Hooper et al., 2010; Argentero and Setti, 2011; Duffy et al., 2015; Missouridou, 2017; Roden-Foreman et al., 2017; Ortega-Campos et al., 2020; Ratrout and Hamdan-Mansour, 2020; Salimi et al., 2020). This may suggest that secondary traumatic stress symptoms are becoming a common character among medical providers working with trauma victims. These findings and those previously mentioned confirm that professionals dealing with the health of others are themselves at risk of its deterioration, especially since STS symptoms may involve other negative consequences of secondary trauma exposure. Such consequences include those occurring at the organizational as well as personal level, such as CF, burnout, feeling overwhelmed, feelings of helplessness, powerlessness, disengagement, lack of work satisfaction, difficulty in enjoying life, interpersonal conflicts, sexual difficulties, and use of drugs and alcohol (Missouridou, 2017; Ratrout and Hamdan-Mansour, 2020). These results confirm that the work of medical providers is extremely stressful.

Severity of STS symptoms were not correlated with profession type (paramedic or nursing), seniority, or gender. They were, however, positively correlated with age, and negatively, although weakly, correlated with workload and the number of hours worked per week.

Analyses confirmed relationships between STS symptoms and both job satisfaction and cognitive processing of trauma, as well as between job satisfaction and cognitive trauma processing. The negative relation between job satisfaction and STS symptoms is consistent with previous studies (Kelly et al., 2015; Kelly and Lefton, 2017; Wang et al., 2020) and confirms that it may play a protective role against the negative consequences of secondary trauma exposure. However an inverse relationship between the variables cannot be ruled out, i.e., that the existing symptoms of STS lead to a decrease in job satisfaction.

Generally, cognitive processing of trauma allows individuals who have experienced traumatic event, either directly or indirectly, to revise their assumptions about themselves and the world. It is also associated with the ability to give the experienced event sense and meaning and thus adapt to a new, changed reality. The negative strategies of regret and denial were positively correlated with STS symptoms, while positive strategies were negatively correlated, which is consistent with previous findings from professionals working with trauma victims (Ogińska-Bulik and Juczyński, 2020). However, the obtained values of correlation coefficients were relatively low. Furthermore, the correlations between STS symptoms and cognitive coping strategies were found to be weaker than the correlations between STS symptoms and job satisfaction.

Job satisfaction was positively correlated with all cognitive coping strategies, more strongly with positive coping strategies. This means that the perceived job satisfaction is conducive to medical professionals undertaking remedial activity, particularly the search for a solution to a problem, its acceptance, and positive cognitive restructuring, as well as, although to a lesser extent, the use of regret and denial. An inverse relationship between the variables is also possible, in that competence in cognitive trauma processing, expressed primarily as using of positive coping strategies, promotes job satisfaction.

The findings showed that cognitive processing of trauma plays a role of mediator in the relation between job satisfaction and symptoms of STS; however, this role varies depending on the coping strategies used. The positive strategy of resolution/acceptance decreases the strength of the correlation between the variables, while the negative strategies of regret and denial increase, albeit slightly, the strength of the relation, acting as a partial suppressor.

Based on these findings we conclude that when medical providers use positive forms of cognitive trauma processing, it decreases the importance of job satisfaction for reducing STS symptoms. In contrast, using negative forms of cognitive trauma processing increases the role of job satisfaction in preventing the negative consequences of indirect trauma exposure.

It is worth mentioning that among the cognitive remedial strategies, positive restructuring, and downward comparison did not play a mediating role. The former correlated, albeit weakly, with STS symptoms, while the latter was not correlated with them. Furthermore, the strategy of downward comparison was generally less important in the process of coping with trauma, and it had the weakest psychometric properties as described in the original version of the CPOTS (Williams et al., 2002).

A mediating role of cognitive remedial strategies is consistent with the literature. For example cognitive processing of trauma played a role of mediator in the correlation between empathy and secondary traumatic stress among therapists, social workers, therapists, and probation officers (Ogińska-Bulik et al., 2020), as well as between trauma exposure and STS symptoms among specialists who work with victims of trauma (Ogińska-Bulik and Juczyński, 2020). In addition, the three cognitive coping strategies resolution/acceptance, downward comparison, and regret, also acted as a mediator in the relationship between negative and positive consequences of trauma in women directly experienced domestic violence (Ogińska-Bulik and Michalska, 2020a).

The present findings confirm the importance of cognitive activity related to trauma and that models of PTSD may also apply to STSD. However, it is important to note that cognitive trauma processing after secondary exposure to trauma may depend on a number of factors, such as the type of traumatic events experienced by clients, the level to which the helpers are cognitively engaged in trauma processing, and the coping resources they possess. Moreover, the negative effects of secondary exposure to trauma do not preclude the possibility of positive consequences revealed by vicarious post-traumatic growth (VPTG). Ongoing research in this area confirms the co-occurrence of STS and VPTG (Manning-Jones et al., 2017; Ogińska-Bulik and Juczyński, 2020). Perceiving positive changes from exposure to the trauma of others can significantly increase the resilience of medical providers to future events.

There are certain limitations to our research. The nature of the study was cross- sectional, and it does not allow concluding about causation effects. Although the study group was large and heterogeneous, there were more men in the paramedic group, while the nursing staff was mostly represented by women. The study did not examine the significance of a traumatic event experienced a direct way, whether work-related or personal. Subjective indicators of indirect trauma exposure were also not included. Furthermore, the Work Satisfaction Scale measures cognitive aspects of job satisfaction but excludes emotional aspects. Finally, we did not examine other coping strategies, including self-care practices, the role of which is stressed as important (Manning-Jones et al., 2016; Molnar et al., 2017). Thus, an inverse correlation between the variables cannot be ruled out, in that job satisfaction mediates the relation between cognitive trauma processing and symptoms of secondary traumatic stress. Despite these limitations, the findings shed important light on the factors determining the negative effects of secondary trauma exposure among healthcare providers, indicating the importance of both job satisfaction and cognitive trauma processing for STS symptoms. Among the strengths of this study is the fact that it was conducted on a large group of individuals which included nurses and rarely studied paramedics. Moreover, using new method to measure STS, developed according to the DSM–5 classification is a further strength.

The findings we describe may inspire further research, which could include emotional aspects of job satisfaction expressed as positive and negative effects, as well as other cognitive trauma processing index, such as disruptions in core beliefs or ruminations about traumatic events happening to patients. It also seems important to examine the role of health professionals’ personal resources, such as self-efficacy or spirituality, which may reduce the severity of STS symptoms. Longitudinal studies with the aim of capturing changes in the intensity of secondary traumatic stress symptoms may also prove valuable.

The current results can be used for practical purposes in the construction of preventive programs aimed at reducing the symptoms of STS. These programs could potentially increase job satisfaction and expand competency in coping with trauma, for instance by using primarily positive coping strategies, especially resolution/acceptance. Moreover, the importance of self-care should not be overlooked as a factor that supports the ability of helping professionals to effectively assist others, as well as potentially improve the quality of their work and personal lives (Molnar et al., 2017; Hricova, 2020).

## Conclusion

Paramedics and nurses are significantly exposed to the occurrence of STS. The results showed significant links between STS symptoms and work satisfaction as well as cognitive processing of trauma. Three cognitive coping strategies play the role of mediators in the relationship between job satisfaction and STS symptoms. The role of individual strategies as mediators in the relationship between job satisfaction and STS varies depending on the strategy used.

## Data Availability Statement

The raw data supporting the conclusions of this article will be made available by the authors, without undue reservation.

## Ethics Statement

The studies involving human participants were reviewed and approved by Opinion of the Bioethics Committee of the National Medical University of Opole No. 59/PI/2020. The patients/participants provided their written informed consent to participate in this study.

## Author Contributions

PG: study design, data collection, interpretation of data, manuscript preparation, and sourcing of funding. NO-B: study design, statistical analysis, interpretation of data, manuscript preparation, and literature review. PM: manuscript preparation and literature review. EK and AS: data collection. All authors contributed to the article and approved the submitted version.

## Conflict of Interest

The authors declare that the research was conducted in the absence of any commercial or financial relationships that could be construed as a potential conflict of interest.

## Publisher’s Note

All claims expressed in this article are solely those of the authors and do not necessarily represent those of their affiliated organizations, or those of the publisher, the editors and the reviewers. Any product that may be evaluated in this article, or claim that may be made by its manufacturer, is not guaranteed or endorsed by the publisher.

## Footnotes

1 The study is part of a larger project. Some of the results of this research were used in another article.

2 Men (*n* = 121) *M* = 31.01, *SD* = 18.43, women (*n* = 80) *M* = 27.65, *SD* = 17.98, *p* > 0.05 among paramedics; men (*n* = 16) *M* = 25.13, *SD* = 17.09, women (*n* = 202) *M* = 32.79, *SD* = 20.89, *p* > 0.05 among nurses.
